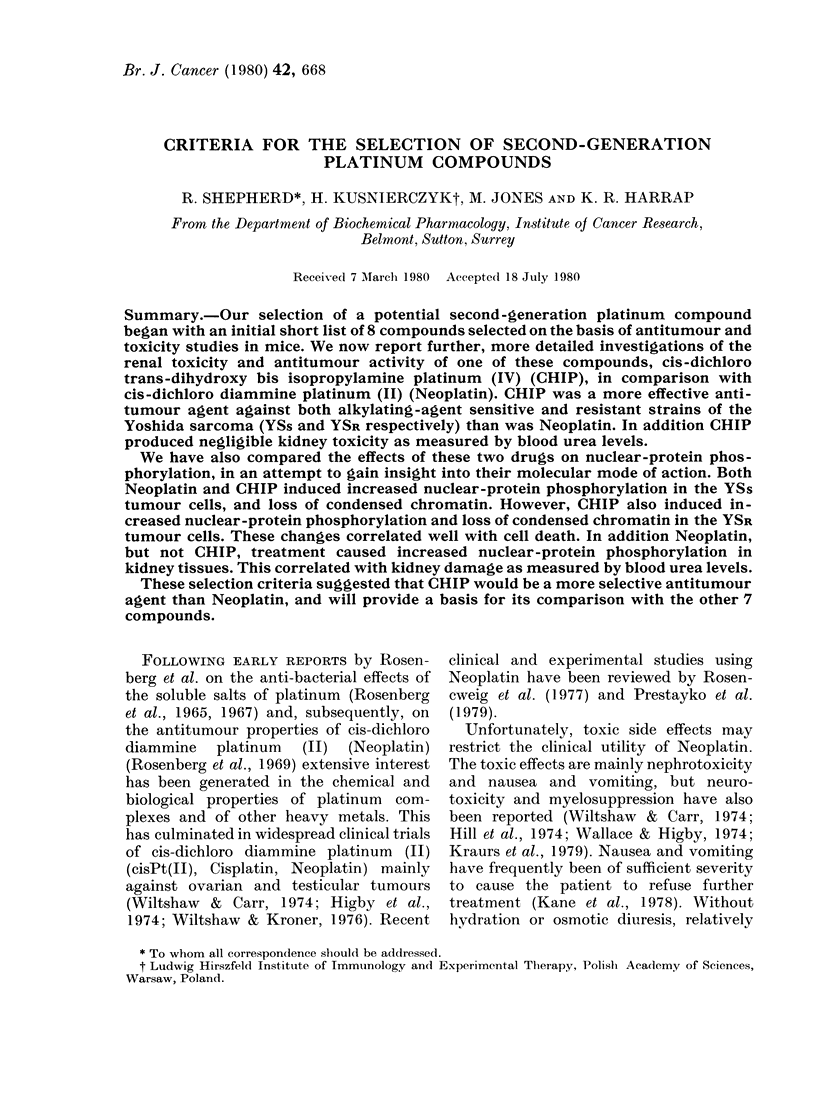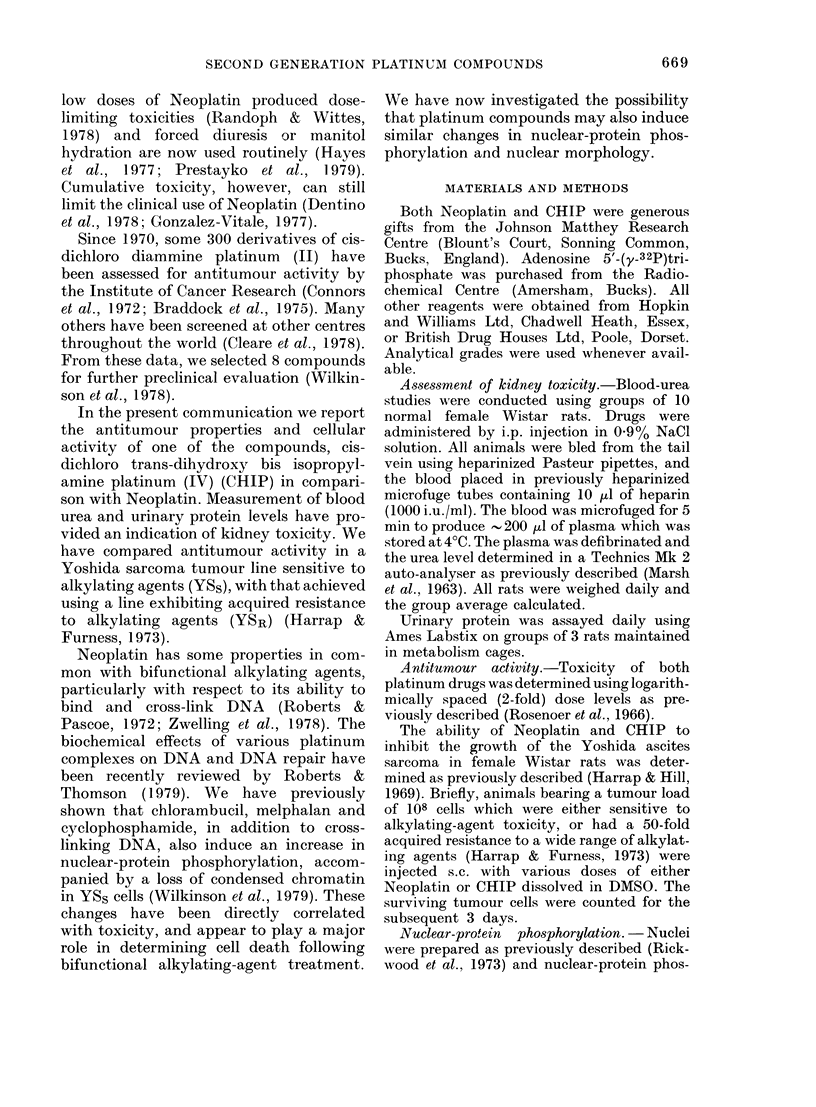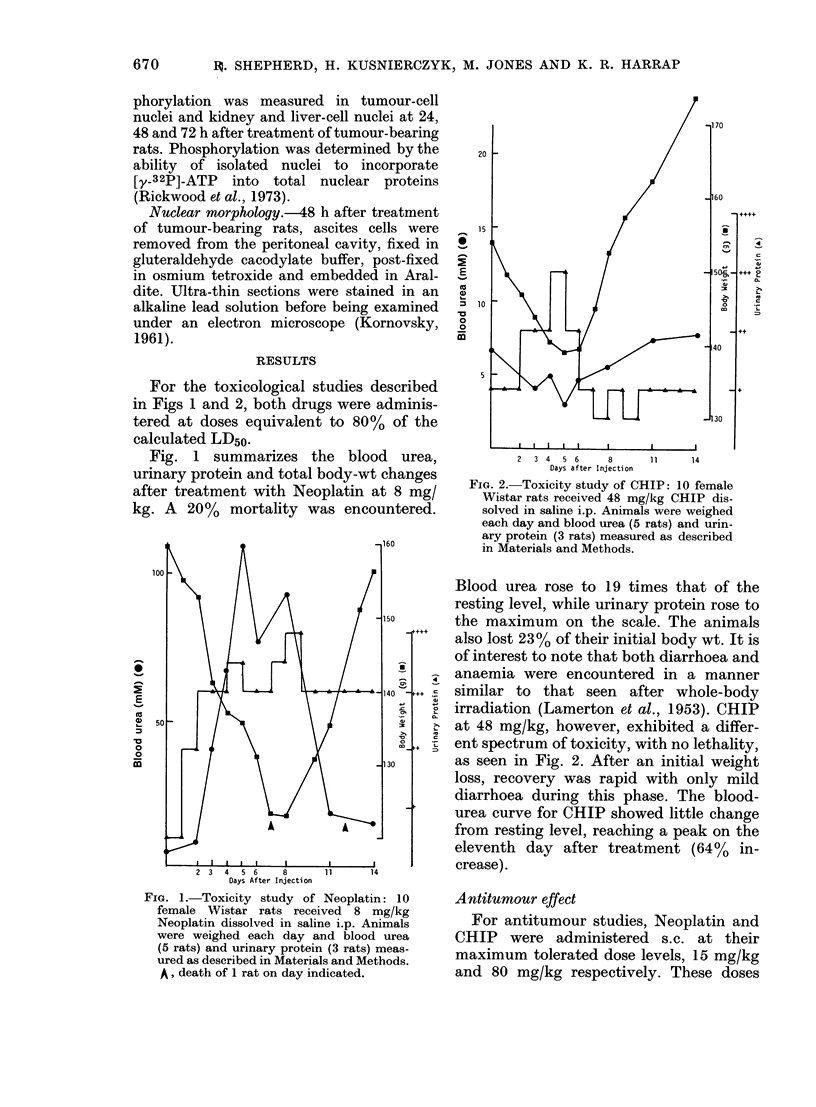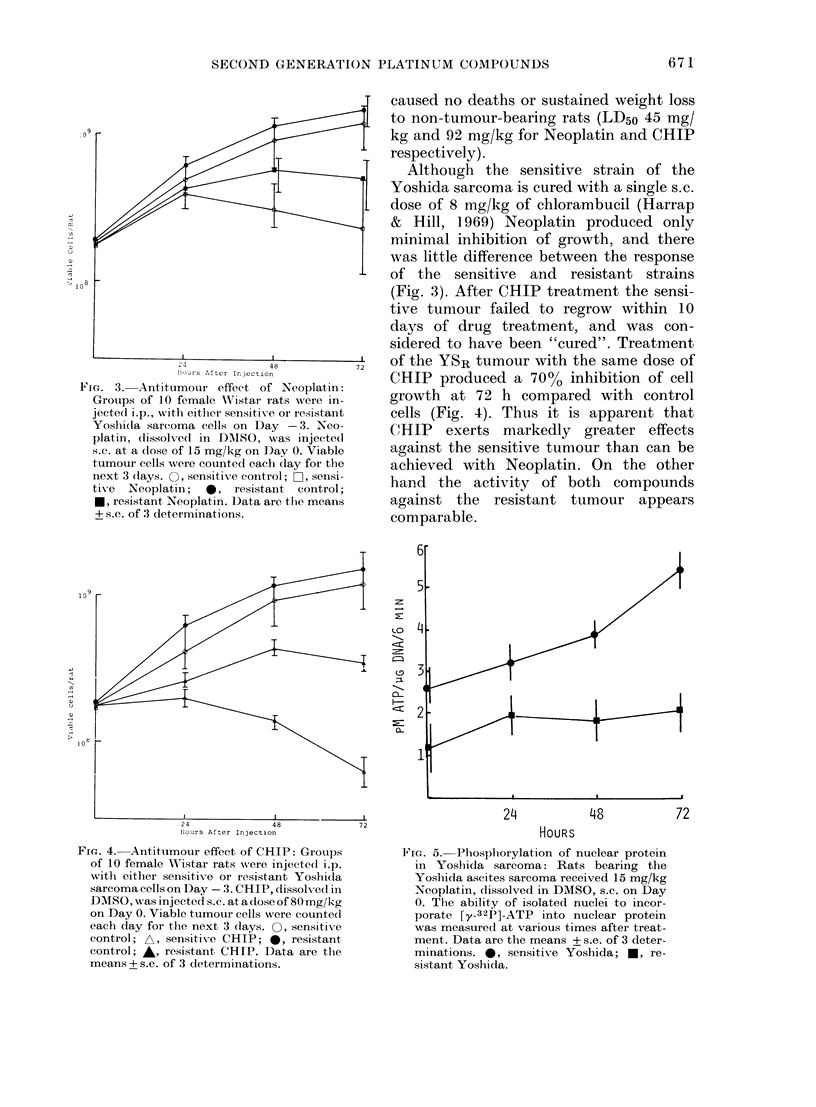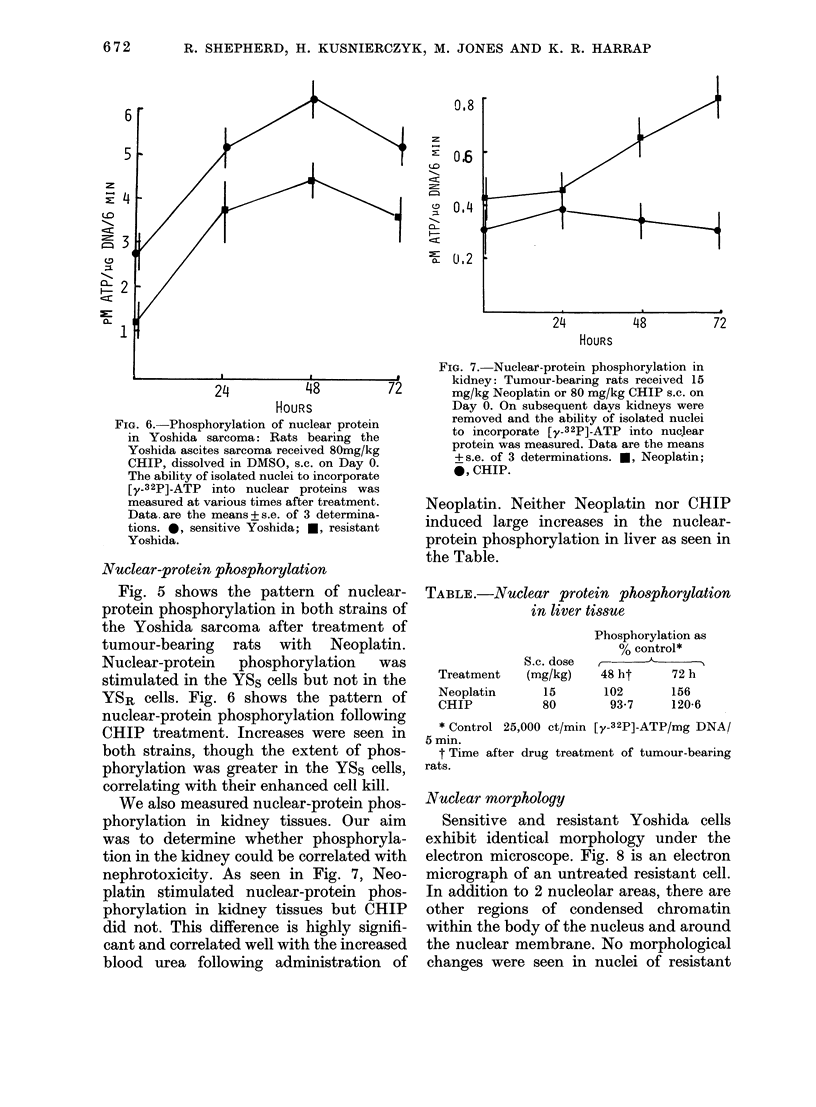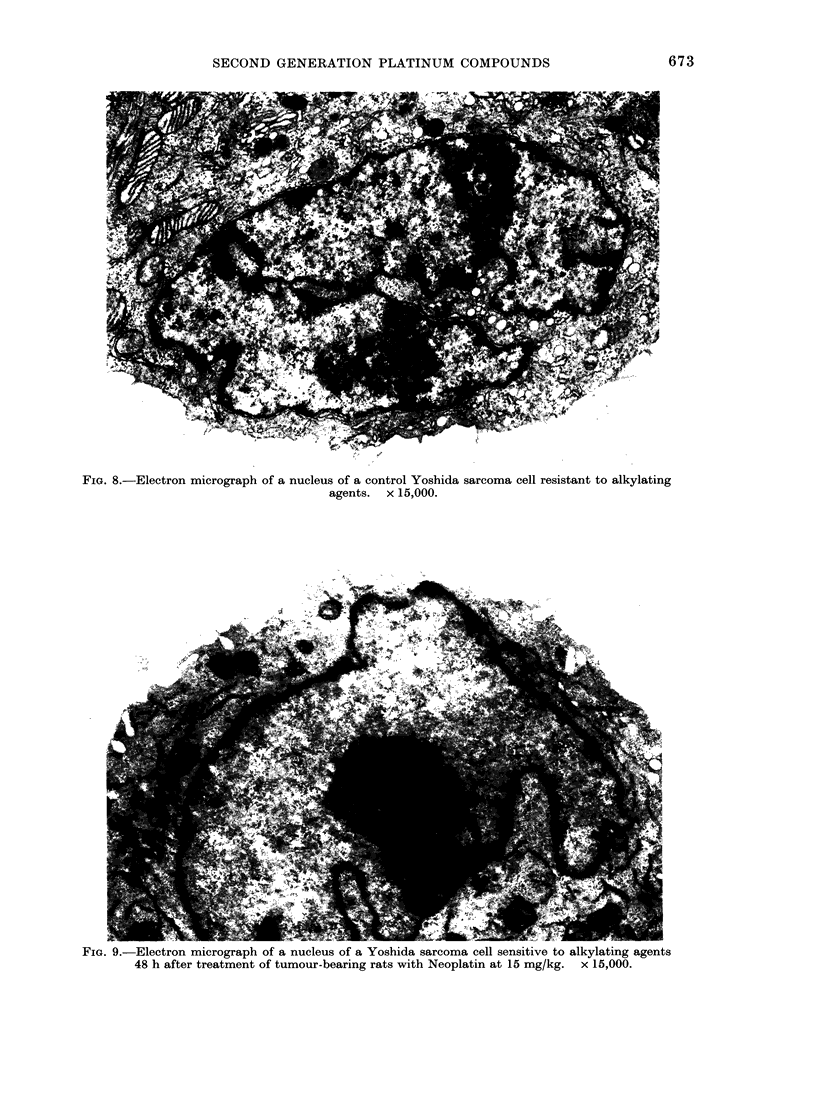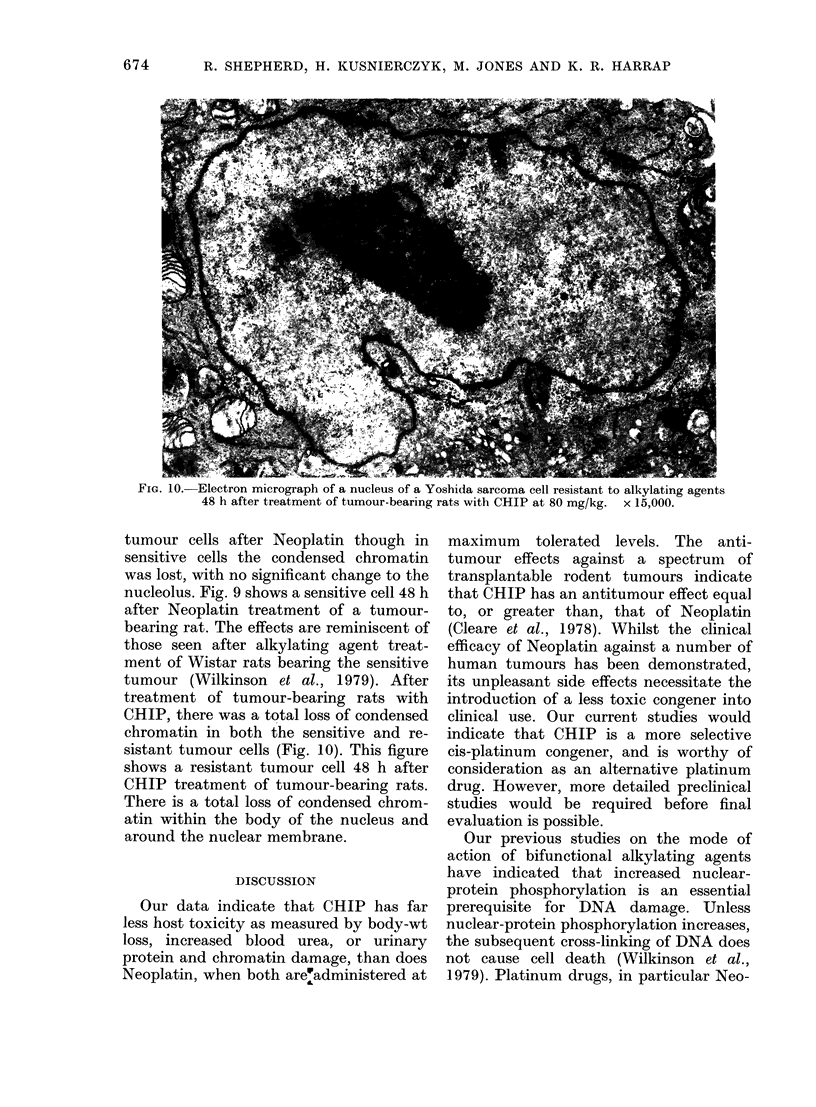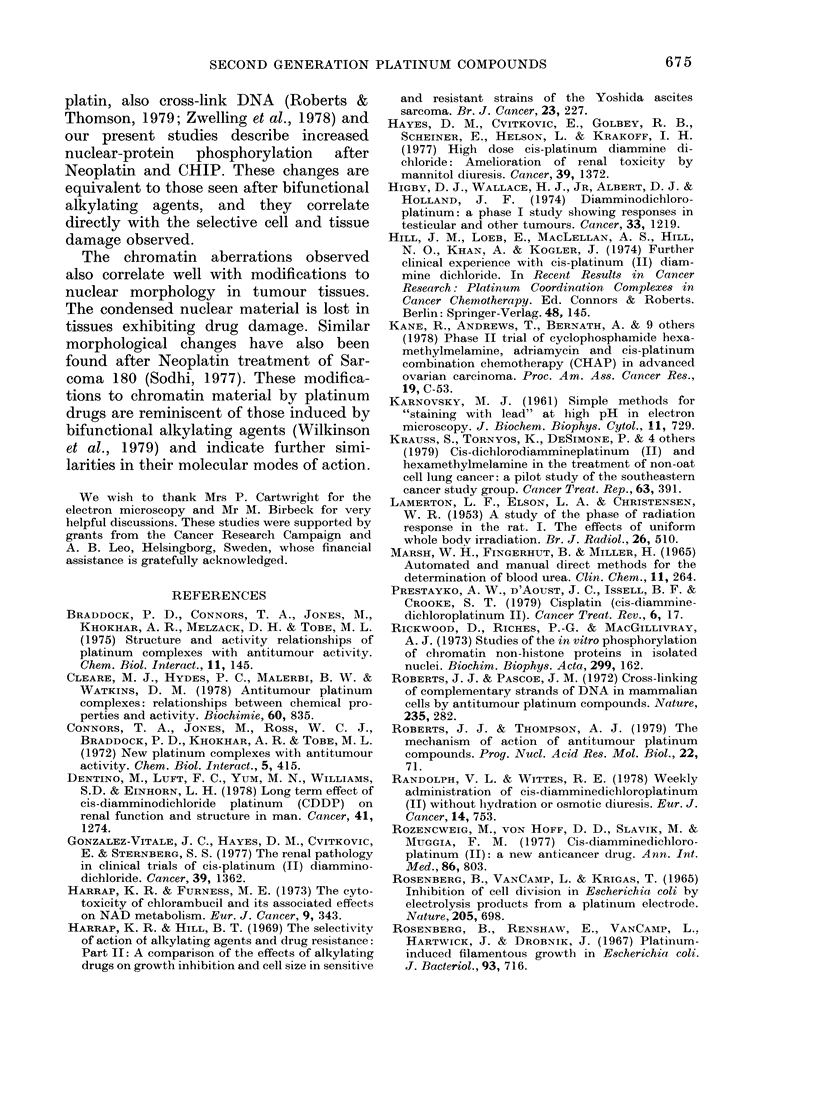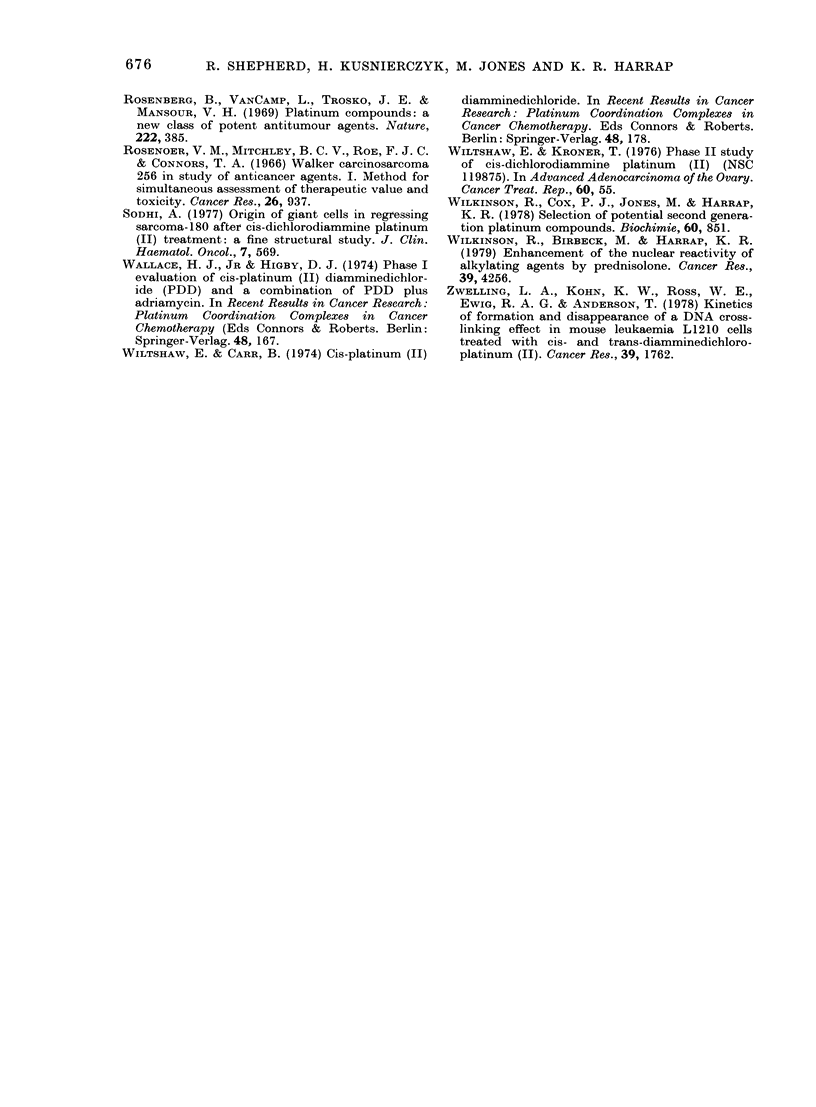# Criteria for the selection of second-generation platinum compounds.

**DOI:** 10.1038/bjc.1980.300

**Published:** 1980-11

**Authors:** R. Shepherd, H. Kusnierczyk, M. Jones, K. R. Harrap

## Abstract

**Images:**


*Br. J. Cancer* (1980) **42**, 668

### CRITERIA FOR THE SELECTION OF SECOND-GENERATION PLATINUM COMPOUNDS

R. SHEPHERD*, H. KUSNIERCZYK†, M. JONES AND K. R. HARRAP

*From the Department of Biochemical Pharmacology, Institute of Cancer Research, Belmont, Sutton, Surrey*

Received 7 March 1980 Accepted 18 July 1980

**Summary.—Our selection of a potential second-generation platinum compound began with an initial short list of 8 compounds selected on the basis of antitumour and toxicity studies in mice. We now report further, more detailed investigations of the renal toxicity and antitumour activity of one of these compounds, cis-dichloro trans-dihydroxy bis isopropylamine platinum (IV) (CHIP), in comparison with cis-dichloro diammine platinum (II) (Neoplatin). CHIP was a more effective antitumour agent against both alkylating-agent sensitive and resistant strains of the Yoshida sarcoma (YSs and YSR respectively) than was Neoplatin. In addition CHIP produced negligible kidney toxicity as measured by blood urea levels.**

**We have also compared the effects of these two drugs on nuclear-protein phosphorylation, in an attempt to gain insight into their molecular mode of action. Both Neoplatin and CHIP induced increased nuclear-protein phosphorylation in the YSs tumour cells, and loss of condensed chromatin. However, CHIP also induced increased nuclear-protein phosphorylation and loss of condensed chromatin in the YSR tumour cells. These changes correlated well with cell death. In addition Neoplatin, but not CHIP, treatment caused increased nuclear-protein phosphorylation in kidney tissues. This correlated with kidney damage as measured by blood urea levels.**

**These selection criteria suggested that CHIP would be a more selective antitumour agent than Neoplatin, and will provide a basis for its comparison with the other 7 compounds.**

FOLLOWING EARLY REPORTS by Rosenberg *et al.* on the anti-bacterial effects of the soluble salts of platinum (Rosenberg *et al.*, 1965, 1967) and, subsequently, on the antitumour properties of cis-dichloro diammine platinum (II) (Neoplatin) (Rosenberg *et al.*, 1969) extensive interest has been generated in the chemical and biological properties of platinum complexes and of other heavy metals. This has culminated in widespread clinical trials of cis-dichloro diammine platinum (II) (cisPt(II), Cisplatin, Neoplatin) mainly against ovarian and testicular tumours (Wiltshaw & Carr, 1974; Higby *et al.*, 1974; Wiltshaw & Kroner, 1976). Recent clinical and experimental studies using Neoplatin have been reviewed by Rosencweig *et al.* (1977) and Prestayko *et al.* (1979).

Unfortunately, toxic side effects may restrict the clinical utility of Neoplatin. The toxic effects are mainly nephrotoxicity and nausea and vomiting, but neurotoxicity and myelosuppression have also been reported (Wiltshaw & Carr, 1974; Hill *et al.*, 1974; Wallace & Higby, 1974; Kraurs *et al.*, 1979). Nausea and vomiting have frequently been of sufficient severity to cause the patient to refuse further treatment (Kane *et al.*, 1978). Without hydration or osmotic diuresis, relatively

* To whom all correspondence should be addressed.
† Ludwig Hirszfeld Institute of Immunology and Experimental Therapy, Polish Academy of Sciences, Warsaw, Poland.

low doses of Neoplatin produced dose-limiting toxicities (Randoph & Wittes, 1978) and forced diuresis or manitol hydration are now used routinely (Hayes *et al.*, 1977; Prestayko *et al.*, 1979). Cumulative toxicity, however, can still limit the clinical use of Neoplatin (Dentino *et al.*, 1978; Gonzalez-Vitale, 1977).

Since 1970, some 300 derivatives of cis-dichloro diammine platinum (II) have been assessed for antitumour activity by the Institute of Cancer Research (Connors *et al.*, 1972; Braddock *et al.*, 1975). Many others have been screened at other centres throughout the world (Cleare *et al.*, 1978). From these data, we selected 8 compounds for further preclinical evaluation (Wilkinson *et al.*, 1978).

In the present communication we report the antitumour properties and cellular activity of one of the compounds, cis-dichloro trans-dihydroxy bis isopropylamine platinum (IV) (CHIP) in comparison with Neoplatin. Measurement of blood urea and urinary protein levels have provided an indication of kidney toxicity. We have compared antitumour activity in a Yoshida sarcoma tumour line sensitive to alkylating agents ($YS_S$), with that achieved using a line exhibiting acquired resistance to alkylating agents ($YS_R$) (Harrap & Furness, 1973).

Neoplatin has some properties in common with bifunctional alkylating agents, particularly with respect to its ability to bind and cross-link DNA (Roberts & Pascoe, 1972; Zwelling *et al.*, 1978). The biochemical effects of various platinum complexes on DNA and DNA repair have been recently reviewed by Roberts & Thomson (1979). We have previously shown that chlorambucil, melphalan and cyclophosphamide, in addition to cross-linking DNA, also induce an increase in nuclear-protein phosphorylation, accompanied by a loss of condensed chromatin in $YS_S$ cells (Wilkinson *et al.*, 1979). These changes have been directly correlated with toxicity, and appear to play a major role in determining cell death following bifunctional alkylating-agent treatment. We have now investigated the possibility that platinum compounds may also induce similar changes in nuclear-protein phosphorylation and nuclear morphology.

### MATERIALS AND METHODS

Both Neoplatin and CHIP were generous gifts from the Johnson Matthey Research Centre (Blount's Court, Sonning Common, Bucks, England). Adenosine 5′-($\gamma$-$^{32}$P)triphosphate was purchased from the Radiochemical Centre (Amersham, Bucks). All other reagents were obtained from Hopkin and Williams Ltd, Chadwell Heath, Essex, or British Drug Houses Ltd, Poole, Dorset. Analytical grades were used whenever available.

*Assessment of kidney toxicity.*—Blood-urea studies were conducted using groups of 10 normal female Wistar rats. Drugs were administered by i.p. injection in 0·9% NaCl solution. All animals were bled from the tail vein using heparinized Pasteur pipettes, and the blood placed in previously heparinized microfuge tubes containing 10 $\mu$l of heparin (1000 i.u./ml). The blood was microfuged for 5 min to produce ~200 $\mu$l of plasma which was stored at 4°C. The plasma was defibrinated and the urea level determined in a Technics Mk 2 auto-analyser as previously described (Marsh *et al.*, 1963). All rats were weighed daily and the group average calculated.

Urinary protein was assayed daily using Ames Labstix on groups of 3 rats maintained in metabolism cages.

*Antitumour activity.*—Toxicity of both platinum drugs was determined using logarithmically spaced (2-fold) dose levels as previously described (Rosenoer *et al.*, 1966).

The ability of Neoplatin and CHIP to inhibit the growth of the Yoshida ascites sarcoma in female Wistar rats was determined as previously described (Harrap & Hill, 1969). Briefly, animals bearing a tumour load of $10^8$ cells which were either sensitive to alkylating-agent toxicity, or had a 50-fold acquired resistance to a wide range of alkylating agents (Harrap & Furness, 1973) were injected s.c. with various doses of either Neoplatin or CHIP dissolved in DMSO. The surviving tumour cells were counted for the subsequent 3 days.

*Nuclear-protein phosphorylation.*—Nuclei were prepared as previously described (Rickwood *et al.*, 1973) and nuclear-protein phosphorylation was measured in tumour-cell nuclei and kidney and liver-cell nuclei at 24, 48 and 72 h after treatment of tumour-bearing rats. Phosphorylation was determined by the ability of isolated nuclei to incorporate [$\gamma$-$^{32}$P]-ATP into total nuclear proteins (Rickwood *et al.*, 1973).

*Nuclear morphology.*—48 h after treatment of tumour-bearing rats, ascites cells were removed from the peritoneal cavity, fixed in gluteraldehyde cacodylate buffer, post-fixed in osmium tetroxide and embedded in Araldite. Ultra-thin sections were stained in an alkaline lead solution before being examined under an electron microscope (Kornovsky, 1961).

### RESULTS

For the toxicological studies described in Figs 1 and 2, both drugs were administered at doses equivalent to 80% of the calculated $LD_{50}$.

Fig. 1 summarizes the blood urea, urinary protein and total body-wt changes after treatment with Neoplatin at 8 mg/kg. A 20% mortality was encountered.

FIG. 1.—Toxicity study of Neoplatin: 10 female Wistar rats received 8 mg/kg Neoplatin dissolved in saline i.p. Animals were weighed each day and blood urea (5 rats) and urinary protein (3 rats) measured as described in Materials and Methods. ▲, death of 1 rat on day indicated.

FIG. 2.—Toxicity study of CHIP: 10 female Wistar rats received 48 mg/kg CHIP dissolved in saline i.p. Animals were weighed each day and blood urea (5 rats) and urinary protein (3 rats) measured as described in Materials and Methods.

Blood urea rose to 19 times that of the resting level, while urinary protein rose to the maximum on the scale. The animals also lost 23% of their initial body wt. It is of interest to note that both diarrhoea and anaemia were encountered in a manner similar to that seen after whole-body irradiation (Lamerton *et al.*, 1953). CHIP at 48 mg/kg, however, exhibited a different spectrum of toxicity, with no lethality, as seen in Fig. 2. After an initial weight loss, recovery was rapid with only mild diarrhoea during this phase. The blood-urea curve for CHIP showed little change from resting level, reaching a peak on the eleventh day after treatment (64% increase).

### *Antitumour effect*

For antitumour studies, Neoplatin and CHIP were administered s.c. at their maximum tolerated dose levels, 15 mg/kg and 80 mg/kg respectively. These doses

FIG. 3.—Antitumour effect of Neoplatin: Groups of 10 female Wistar rats were injected i.p., with either sensitive or resistant Yoshida sarcoma cells on Day −3. Neoplatin, dissolved in DMSO, was injected s.c. at a dose of 15 mg/kg on Day 0. Viable tumour cells were counted each day for the next 3 days. ○, sensitive control; □, sensitive Neoplatin; ●, resistant control; ■, resistant Neoplatin. Data are the means ± s.e. of 3 determinations.

caused no deaths or sustained weight loss to non-tumour-bearing rats ($LD_{50}$ 45 mg/kg and 92 mg/kg for Neoplatin and CHIP respectively).

Although the sensitive strain of the Yoshida sarcoma is cured with a single s.c. dose of 8 mg/kg of chlorambucil (Harrap & Hill, 1969) Neoplatin produced only minimal inhibition of growth, and there was little difference between the response of the sensitive and resistant strains (Fig. 3). After CHIP treatment the sensitive tumour failed to regrow within 10 days of drug treatment, and was considered to have been "cured". Treatment of the $YS_R$ tumour with the same dose of CHIP produced a 70% inhibition of cell growth at 72 h compared with control cells (Fig. 4). Thus it is apparent that CHIP exerts markedly greater effects against the sensitive tumour than can be achieved with Neoplatin. On the other hand the activity of both compounds against the resistant tumour appears comparable.

FIG. 4.—Antitumour effect of CHIP: Groups of 10 female Wistar rats were injected i.p. with either sensitive or resistant Yoshida sarcoma cells on Day −3. CHIP, dissolved in DMSO, was injected s.c. at a dose of 80 mg/kg on Day 0. Viable tumour cells were counted each day for the next 3 days. ○, sensitive control; △, sensitive CHIP; ●, resistant control; ▲, resistant CHIP. Data are the means ± s.e. of 3 determinations.

FIG. 5.—Phosphorylation of nuclear protein in Yoshida sarcoma: Rats bearing the Yoshida ascites sarcoma received 15 mg/kg Neoplatin, dissolved in DMSO, s.c. on Day 0. The ability of isolated nuclei to incorporate [$\gamma$-$^{32}$P]-ATP into nuclear protein was measured at various times after treatment. Data are the means ± s.e. of 3 determinations. ●, sensitive Yoshida; ■, resistant Yoshida.

FIG. 6.—Phosphorylation of nuclear protein in Yoshida sarcoma: Rats bearing the Yoshida ascites sarcoma received 80mg/kg CHIP, dissolved in DMSO, s.c. on Day 0. The ability of isolated nuclei to incorporate [$\gamma$-$^{32}$P]-ATP into nuclear proteins was measured at various times after treatment. Data are the means ± s.e. of 3 determinations. ●, sensitive Yoshida; ■, resistant Yoshida.

*Nuclear-protein phosphorylation*

Fig. 5 shows the pattern of nuclear-protein phosphorylation in both strains of the Yoshida sarcoma after treatment of tumour-bearing rats with Neoplatin. Nuclear-protein phosphorylation was stimulated in the $YS_S$ cells but not in the $YS_R$ cells. Fig. 6 shows the pattern of nuclear-protein phosphorylation following CHIP treatment. Increases were seen in both strains, though the extent of phosphorylation was greater in the $YS_S$ cells, correlating with their enhanced cell kill.

We also measured nuclear-protein phosphorylation in kidney tissues. Our aim was to determine whether phosphorylation in the kidney could be correlated with nephrotoxicity. As seen in Fig. 7, Neoplatin stimulated nuclear-protein phosphorylation in kidney tissues but CHIP did not. This difference is highly significant and correlated well with the increased blood urea following administration of Neoplatin. Neither Neoplatin nor CHIP induced large increases in the nuclear-protein phosphorylation in liver as seen in the Table.

FIG. 7.—Nuclear-protein phosphorylation in kidney: Tumour-bearing rats received 15 mg/kg Neoplatin or 80 mg/kg CHIP s.c. on Day 0. On subsequent days kidneys were removed and the ability of isolated nuclei to incorporate [$\gamma$-$^{32}$P]-ATP into nuclear protein was measured. Data are the means ± s.e. of 3 determinations. ■, Neoplatin; ●, CHIP.

TABLE.—*Nuclear protein phosphorylation in liver tissue*

| Treatment | S.c. dose (mg/kg) | Phosphorylation as % control* 48 h† | 72 h |
|---|---|---|---|
| Neoplatin | 15 | 102 | 156 |
| CHIP | 80 | 93·7 | 120·6 |

* Control 25,000 ct/min [$\gamma$-$^{32}$P]-ATP/mg DNA/5 min.

† Time after drug treatment of tumour-bearing rats.

*Nuclear morphology*

Sensitive and resistant Yoshida cells exhibit identical morphology under the electron microscope. Fig. 8 is an electron micrograph of an untreated resistant cell. In addition to 2 nucleolar areas, there are other regions of condensed chromatin within the body of the nucleus and around the nuclear membrane. No morphological changes were seen in nuclei of resistant

FIG. 8.—Electron micrograph of a nucleus of a control Yoshida sarcoma cell resistant to alkylating agents. ×15,000.

FIG. 9.—Electron micrograph of a nucleus of a Yoshida sarcoma cell sensitive to alkylating agents 48 h after treatment of tumour-bearing rats with Neoplatin at 15 mg/kg. ×15,000.

FIG. 10.—Electron micrograph of a nucleus of a Yoshida sarcoma cell resistant to alkylating agents 48 h after treatment of tumour-bearing rats with CHIP at 80 mg/kg. × 15,000.

tumour cells after Neoplatin though in sensitive cells the condensed chromatin was lost, with no significant change to the nucleolus. Fig. 9 shows a sensitive cell 48 h after Neoplatin treatment of a tumour-bearing rat. The effects are reminiscent of those seen after alkylating agent treatment of Wistar rats bearing the sensitive tumour (Wilkinson *et al.*, 1979). After treatment of tumour-bearing rats with CHIP, there was a total loss of condensed chromatin in both the sensitive and resistant tumour cells (Fig. 10). This figure shows a resistant tumour cell 48 h after CHIP treatment of tumour-bearing rats. There is a total loss of condensed chromatin within the body of the nucleus and around the nuclear membrane.

### DISCUSSION

Our data indicate that CHIP has far less host toxicity as measured by body-wt loss, increased blood urea, or urinary protein and chromatin damage, than does Neoplatin, when both are administered at maximum tolerated levels. The antitumour effects against a spectrum of transplantable rodent tumours indicate that CHIP has an antitumour effect equal to, or greater than, that of Neoplatin (Cleare *et al.*, 1978). Whilst the clinical efficacy of Neoplatin against a number of human tumours has been demonstrated, its unpleasant side effects necessitate the introduction of a less toxic congener into clinical use. Our current studies would indicate that CHIP is a more selective cis-platinum congener, and is worthy of consideration as an alternative platinum drug. However, more detailed preclinical studies would be required before final evaluation is possible.

Our previous studies on the mode of action of bifunctional alkylating agents have indicated that increased nuclear-protein phosphorylation is an essential prerequisite for DNA damage. Unless nuclear-protein phosphorylation increases, the subsequent cross-linking of DNA does not cause cell death (Wilkinson *et al.*, 1979). Platinum drugs, in particular Neoplatin, also cross-link DNA (Roberts & Thomson, 1979; Zwelling *et al.*, 1978) and our present studies describe increased nuclear-protein phosphorylation after Neoplatin and CHIP. These changes are equivalent to those seen after bifunctional alkylating agents, and they correlate directly with the selective cell and tissue damage observed.

The chromatin aberrations observed also correlate well with modifications to nuclear morphology in tumour tissues. The condensed nuclear material is lost in tissues exhibiting drug damage. Similar morphological changes have also been found after Neoplatin treatment of Sarcoma 180 (Sodhi, 1977). These modifications to chromatin material by platinum drugs are reminiscent of those induced by bifunctional alkylating agents (Wilkinson *et al.*, 1979) and indicate further similarities in their molecular modes of action.

We wish to thank Mrs P. Cartwright for the electron microscopy and Mr M. Birbeck for very helpful discussions. These studies were supported by grants from the Cancer Research Campaign and A. B. Leo, Helsingborg, Sweden, whose financial assistance is gratefully acknowledged.

### REFERENCES

Braddock, P. D., Connors, T. A., Jones, M., Khokhar, A. R., Melzack, D. H. & Tobe, M. L. (1975) Structure and activity relationships of platinum complexes with antitumour activity. *Chem. Biol. Interact.*, **11**, 145.

Cleare, M. J., Hydes, P. C., Malerbi, B. W. & Watkins, D. M. (1978) Antitumour platinum complexes: relationships between chemical properties and activity. *Biochimie*, **60**, 835.

Connors, T. A., Jones, M., Ross, W. C. J., Braddock, P. D., Khokhar, A. R. & Tobe, M. L. (1972) New platinum complexes with antitumour activity. *Chem. Biol. Interact.*, **5**, 415.

Dentino, M., Luft, F. C., Yum, M. N., Williams, S.D. & Einhorn, L. H. (1978) Long term effect of cis-diamminodichloride platinum (CDDP) on renal function and structure in man. *Cancer*, **41**, 1274.

Gonzalez-Vitale, J. C., Hayes, D. M., Cvitkovic, E. & Sternberg, S. S. (1977) The renal pathology in clinical trials of cis-platinum (II) diamminodichloride. *Cancer*, **39**, 1362.

Harrap, K. R. & Furness, M. E. (1973) The cytotoxicity of chlorambucil and its associated effects on NAD metabolism. *Eur. J. Cancer*, **9**, 343.

Harrap, K. R. & Hill, B. T. (1969) The selectivity of action of alkylating agents and drug resistance: Part II: A comparison of the effects of alkylating drugs on growth inhibition and cell size in sensitive and resistant strains of the Yoshida ascites sarcoma. *Br. J. Cancer*, **23**, 227.

Hayes, D. M., Cvitkovic, E., Golbey, R. B., Scheiner, E., Helson, L. & Krakoff, I. H. (1977) High dose cis-platinum diammine dichloride: Amelioration of renal toxicity by mannitol diuresis. *Cancer*, **39**, 1372.

Higby, D. J., Wallace, H. J., Jr, Albert, D. J. & Holland, J. F. (1974) Diamminodichloroplatinum: a phase I study showing responses in testicular and other tumours. *Cancer*, **33**, 1219.

Hill, J. M., Loeb, E., MacLellan, A. S., Hill, N. O., Khan, A. & Kogler, J. (1974) Further clinical experience with cis-platinum (II) diammine dichloride. In *Recent Results in Cancer Research: Platinum Coordination Complexes in Cancer Chemotherapy*. Ed. Connors & Roberts. Berlin: Springer-Verlag. **48**, 145.

Kane, R., Andrews, T., Bernath, A. & 9 others (1978) Phase II trial of cyclophosphamide hexamethylmelamine, adriamycin and cis-platinum combination chemotherapy (CHAP) in advanced ovarian carcinoma. *Proc. Am. Ass. Cancer Res.*, **19**, C-53.

Karnovsky, M. J. (1961) Simple methods for "staining with lead" at high pH in electron microscopy. *J. Biochem. Biophys. Cytol.*, **11**, 729.

Krauss, S., Tornyos, K., DeSimone, P. & 4 others (1979) Cis-dichlorodiammineplatinum (II) and hexamethylmelamine in the treatment of non-oat cell lung cancer: a pilot study of the southeastern cancer study group. *Cancer Treat. Rep.*, **63**, 391.

Lamerton, L. F., Elson, L. A. & Christensen, W. R. (1953) A study of the phase of radiation response in the rat. I. The effects of uniform whole body irradiation. *Br. J. Radiol.*, **26**, 510.

Marsh, W. H., Fingerhut, B. & Miller, H. (1965) Automated and manual direct methods for the determination of blood urea. *Clin. Chem.*, **11**, 264.

Prestayko, A. W., d'Aoust, J. C., Issell, B. F. & Crooke, S. T. (1979) Cisplatin (cis-diamminedichloroplatinum II). *Cancer Treat. Rev.*, **6**, 17.

Rickwood, D., Riches, P.-G. & MacGillivray, A. J. (1973) Studies of the *in vitro* phosphorylation of chromatin non-histone proteins in isolated nuclei. *Biochim. Biophys. Acta*, **299**, 162.

Roberts, J. J. & Pascoe, J. M. (1972) Cross-linking of complementary strands of DNA in mammalian cells by antitumour platinum compounds. *Nature*, **235**, 282.

Roberts, J. J. & Thompson, A. J. (1979) The mechanism of action of antitumour platinum compounds. *Prog. Nucl. Acid Res. Mol. Biol.*, **22**, 71.

Randolph, V. L. & Wittes, R. E. (1978) Weekly administration of cis-diamminedichloroplatinum (II) without hydration or osmotic diuresis. *Eur. J. Cancer*, **14**, 753.

Rozencweig, M., von Hoff, D. D., Slavik, M. & Muggia, F. M. (1977) Cis-diamminedichloroplatinum (II): a new anticancer drug. *Ann. Int. Med.*, **86**, 803.

Rosenberg, B., VanCamp, L. & Krigas, T. (1965) Inhibition of cell division in *Escherichia coli* by electrolysis products from a platinum electrode. *Nature*, **205**, 698.

Rosenberg, B., Renshaw, E., VanCamp, L., Hartwick, J. & Drobnik, J. (1967) Platinum-induced filamentous growth in *Escherichia coli*. *J. Bacteriol.*, **93**, 716.

ROSENBERG, B., VANCAMP, L., TROSKO, J. E. & MANSOUR, V. H. (1969) Platinum compounds: a new class of potent antitumour agents. *Nature*, **222**, 385.

ROSENOER, V. M., MITCHLEY, B. C. V., ROE, F. J. C. & CONNORS, T. A. (1966) Walker carcinosarcoma 256 in study of anticancer agents. I. Method for simultaneous assessment of therapeutic value and toxicity. *Cancer Res.*, **26**, 937.

SODHI, A. (1977) Origin of giant cells in regressing sarcoma-180 after cis-dichlorodiammine platinum (II) treatment: a fine structural study. *J. Clin. Haematol. Oncol.*, **7**, 569.

WALLACE, H. J., JR & HIGBY, D. J. (1974) Phase I evaluation of cis-platinum (II) diamminedichloride (PDD) and a combination of PDD plus adriamycin. In *Recent Results in Cancer Research: Platinum Coordination Complexes in Cancer Chemotherapy* (Eds Connors & Roberts. Berlin: Springer-Verlag. **48**, 167.

WILTSHAW, E. & CARR, B. (1974) Cis-platinum (II) diamminedichloride. In *Recent Results in Cancer Research: Platinum Coordination Complexes in Cancer Chemotherapy*. Eds Connors & Roberts. Berlin: Springer-Verlag. **48**, 178.

WILTSHAW, E. & KRONER, T. (1976) Phase II study of cis-dichlorodiammine platinum (II) (NSC 119875). In *Advanced Adenocarcinoma of the Ovary. Cancer Treat. Rep.*, **60**, 55.

WILKINSON, R., COX, P. J., JONES, M. & HARRAP, K. R. (1978) Selection of potential second generation platinum compounds. *Biochimie*, **60**, 851.

WILKINSON, R., BIRBECK, M. & HARRAP, K. R. (1979) Enhancement of the nuclear reactivity of alkylating agents by prednisolone. *Cancer Res.*, **39**, 4256.

ZWELLING, L. A., KOHN, K. W., ROSS, W. E., EWIG, R. A. G. & ANDERSON, T. (1978) Kinetics of formation and disappearance of a DNA cross-linking effect in mouse leukaemia L1210 cells treated with cis- and trans-diamminedichloroplatinum (II). *Cancer Res.*, **39**, 1762.